# Microbiome–metabolome dynamics associated with impaired glucose control and responses to lifestyle changes

**DOI:** 10.1038/s41591-025-03642-6

**Published:** 2025-04-08

**Authors:** Hao Wu, Bomin Lv, Luqian Zhi, Yikai Shao, Xinyan Liu, Matthias Mitteregger, Rima Chakaroun, Valentina Tremaroli, Stanley L. Hazen, Ru Wang, Göran Bergström, Fredrik Bäckhed

**Affiliations:** 1 Center for Obesity and Hernia Surgery, Department of General Surgery, Huashan Hospital, and State Key Laboratory of Genetic Engineering, Fudan Microbiome Center, Human Phenome Institute, Fudan University, Shanghai, China; 2 The Wallenberg Laboratory, Department of Molecular and Clinical Medicine, Sahlgrenska Academy, University of Gothenburg, Gothenburg, Sweden; 3 Medical Department III – Endocrinology, Nephrology, Rheumatology, University of Leipzig Medical Center, Leipzig, Germany; 4 Department of Cardiovascular & Metabolic Sciences, Lerner Research Institute, Cleveland, OH USA; 5 Center for Microbiome and Human Health, Cleveland Clinic, Cleveland, OH USA; 6 Department of Cardiovascular Medicine, Heart, Vascular and Thoracic Institute, Cleveland Clinic, Cleveland, OH USA; 7 School of Kinesiology, Key Laboratory of Exercise and Health Sciences of Ministry of Education, Shanghai University of Sport, Shanghai, China; 8 Department of Clinical Physiology, Region Västra Götaland, Sahlgrenska University Hospital, Gothenburg, Sweden

**Keywords:** Microbial communities, Diabetes, Metabolomics

## Abstract

Type 2 diabetes (T2D) is a complex disease shaped by genetic and environmental factors, including the gut microbiome. Recent research revealed pathophysiological heterogeneity and distinct subgroups in both T2D and prediabetes, prompting exploration of personalized risk factors. Using metabolomics in two Swedish cohorts (*n* = 1,167), we identified over 500 blood metabolites associated with impaired glucose control, with approximately one-third linked to an altered gut microbiome. Our findings identified metabolic disruptions in microbiome–metabolome dynamics as potential mediators of compromised glucose homeostasis, as illustrated by the potential interactions between *Hominifimenecus microfluidus* and *Blautia wexlerae* via hippurate. Short-term lifestyle changes, for example, diet and exercise, modulated microbiome-associated metabolites in a lifestyle-specific manner. This study suggests that the microbiome–metabolome axis is a modifiable target for T2D management, with optimal health benefits achievable through a combination of lifestyle modifications.

## Abstract (web summary)

A map of microbiome–metabolome dynamics in people with type 2 diabetes identifies over 500 blood metabolites associated with impaired glucose control, with approximately one-third linked to an altered gut microbiome.

## Main

Type 2 diabetes (T2D) is influenced by both genetic and environmental factors^1–3^; recent research suggested that it may consist of distinct pathophysiological subgroups^4–6^. Thus, there is a growing interest in using multi-omic approaches to identify personalized risk factors for T2D^7,8^ and related comorbidities^9^, such as obesity^10^, acute coronary syndrome (ACS)^11^, ischemic heart disease^12^ and heart failure (HF)^13^. The circulating metabolome originates from the compound effects of diet, host and microbiome^14–16^, which through dynamic interactions contribute to the pathogenesis, development and treatment responses in cardiometabolic diseases^17–20^. Approximately 70% of incident T2D cases can be attributed to suboptimal diet^21^, which affects the gut microbiota^22^; in turn, the microbiota contributes to 10–15% of the variation in fasting circulating metabolite levels^14,16,23^. Accordingly, circulating metabolites may reflect changes in the gut microbiome^24^, with altered microbiome–metabolome dynamics contributing to T2D and prediabetes phenotypes^7,8,25–27^.

To identify microbial metabolites linked to phenotype heterogeneity in prediabetes and T2D, we performed metabolomic profiling in two Swedish cohorts, spanning from normal glucose tolerance (NGT) to treatment-naive T2D. To inform potential therapeutic strategies for addressing glucose intolerance, we also assessed the impact of short-term lifestyle interventions, either diet or exercise, on T2D-related metabolites. We additionally constructed an open-access web server to facilitate metabolome data exploration, visualization and meta-analysis (https://omicsdata.org/Apps/IGT_metabolome/).

## Results

### Determinants of plasma metabolites in individuals with impaired glucose control

**Fig. 1:**
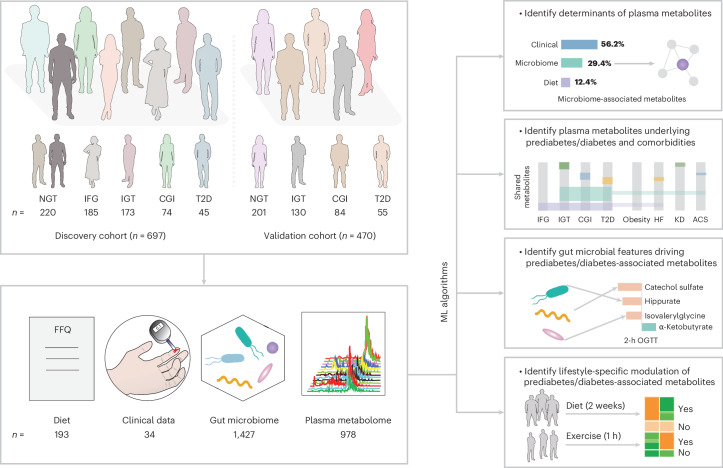
Study design and data collection strategy. FBG and OGTT were used to screen individuals with varying degree of glucose intolerance. The GBDT algorithm was used to predict plasma metabolites based on collected data from the FFQ, clinical tests and gut microbiome profiling. *n* indicates the sample size for the two cohorts, or the number of features in the diet, clinical, gut microbiome and plasma metabolome datasets.

Metabolomic profiling was performed on plasma samples collected from individuals (aged 50–64 years) with prediabetes, treatment-naive T2D and controls, who were included in the impaired glucose tolerance (IGT) (*n* = 697) and Swedish CArdioPulmonary bioImage Study (SCAPIS) (*n* = 470) cohorts from Sweden^25^, serving as discovery and validation cohorts, respectively (Fig. 1). In the discovery cohort, 220 individuals had NGT, 185 had isolated impaired fasting glucose (IFG), 173 had isolated IGT, 74 had combined glucose intolerance (CGI) and 45 had screen-detected T2D based on fasting glucose levels or oral glucose tolerance test (OGTT). In the validation cohort, 201 individuals had NGT, 130 had isolated IGT, 84 had CGI and 55 had T2D. As 364 of 477 individuals (76.3%) with prediabetes and T2D in the discovery cohort were overweight or obese (body mass index (BMI ≥ 25)), the NGT group was BMI-matched with the IGT group in the validation cohort (Supplementary Table 1) to partly mitigate the potential confounding effects of overweight and obesity. The detailed clinical characteristics of both cohorts can be found in Supplementary Table 1. A total of 978 plasma metabolites, primarily derived from amino acids (22.1%) and lipid (45.4%) metabolism, were measured and annotated (Supplementary Table 2).

**Fig. 2:**
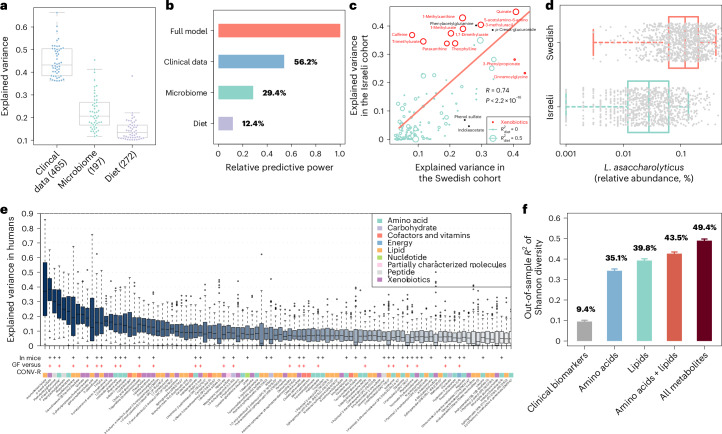
Robust prediction of microbiome-associated metabolites. **a**, Box and swarm plots illustrating the explained variance of the top 50 significantly predicted metabolites from clinical data, microbiome and diet using the GBDT algorithm in the discovery cohort. The *P* values were estimated based on 1,000 iterations of bootstrapping; the number of metabolites with significant predictions in each group are shown in parentheses (Wald test, two-sided *P*_adj_ < 0.1). The boxes show the median (line), the 25th/75th percentiles (box) and 1.5 times the interquartile range (IQR) (whiskers). **b**, Comparison of the explained variance according to each feature group, with the full model incorporating all features. **c**, Explained variance for the 197 predicted microbiome-associated metabolites in the Swedish versus Israeli cohorts. Fifteen influential outliers (with biochemical names labeled) were identified using Cook’s distance (larger than three times the mean Cook’s distance) based on a linear regression model (raw two-sided *P* < 2.2 × 10^−16^). The circle sizes are proportional to the explained variance according to diet in the Israel cohort. **d**, Relative abundances of *L. asaccharolyticus* in the Israeli versus Swedish cohorts (*n* = 969 and *n* = 1,167, respectively). The boxes show the median (line), the 25th/75th percentiles (box) and 1.5 times the IQR (whiskers). **e**, The top 100 metabolites, ranked according to the explained variance from microbiome data, and their differences in plasma levels between CONV-R and GF mice. The symbols (+) in black and red indicate whether a metabolite was detected in mice and whether it significantly differed between the two groups (Wilcoxon rank-sum test, two-sided *P*_adj_ < 0.1), respectively. Labelled metabolite names (*) indicate Metabolon-anotated IDs requiring validation. The boxes show the median (line), the 25th/75th percentiles (box) and 1.5 times the IQR (whiskers). **f**, Explained variance (mean out-of-sample *R*²) of the Shannon index according to clinical biomarkers, amino acids, lipids or all metabolites was assessed using random forest regression (*n* = 697 samples), with tenfold cross-validation repeated ten times (*n* = 100 repeats; shown as the mean ± s.e.m.).

**Extended Data Fig. 1:**
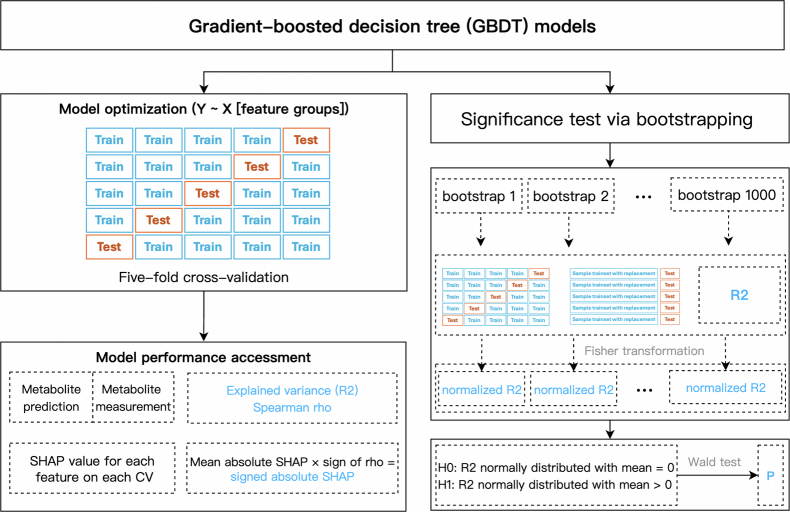
Flowchart showing metabolites prediction procedures using GBDT models over the clinical, microbiome, and diet feature groups. GBDT, gradient-boosted decision tree.

While clinical phenotypes, microbiome and diet have been linked to the blood metabolome in healthy individuals from Israel^14^, it is important to explore whether these factors also applied to the Swedish cohort, including those with prediabetes and T2D. To this aim, we used the same analytical strategy, that is, the gradient-boosted decision trees (GBDT) algorithm^14^ (Methods and Extended Data Fig. 1). We evaluated the relative predictive power of these three feature groups, including 34 clinical biomarkers (Supplementary Table 1), 1,427 metagenomic species (MGSs) (Supplementary Table 3)^25^ and 193 dietary variables based on MiniMeal-Q^28,29^, a validated web-based interactive food frequency questionnaire (FFQ) (Supplementary Table 4), respectively, for each circulating metabolite measured in the Swedish IGT cohort, ranging from normal glucose control to treatment-naive T2D. In total, we observed that 645 of 978 (65.9%) metabolites were significantly associated with at least one feature group (Supplementary Table 5; Wald test, *P*_adj_ < 0.1). In particular, we found that GBDT models explained a median and maximum explained variance of 13.6% and 66.3%, respectively, to predict the circulating levels of each metabolite with the clinical data (465 associated metabolites in total), 7.8% and 47.2%, respectively, with the microbiome data (197 metabolites), and 1.3% and 38.3%, respectively, with the diet data (272 metabolites) (Fig. 2a and Supplementary Table 5). The relative predictive power of these three factors over the whole metabolome, calculated based on new GBDT models to predict the principal metabolomics components, was 56.2%, 29.4% and 12.4% of the full model for clinical, microbiome and diet data (Fig. 2b), respectively. These findings show that potential determinants persist in prediabetes and T2D, with the gut microbiome alone accounting for nearly one-third of blood metabolite variance—twice that measured in healthy individuals^14–16,23^.

### Robust predictions of microbiome-associated metabolites

We next used five distinct approaches to predict and validate the 197 microbiome-associated metabolites identified (Fig. 2a and Supplementary Table 5): (1) we evaluated the impact of distinct metagenomics pipelines, including reference-free canopy clustering^25^, the reference-based Kraken 2 (ref. ^30^) and the lineage-specific marker-gene-based MetaPhlAn 4 (ref. ^31^) to predict the microbiome-associated metabolites; (2) we used two machine learning (ML) methods, that is, GBDT and random forest, to establish microbiome–metabolome associations based on the same MGSs^25^; (3) we used the same ML algorithms to link metabolites to the Kyoto Encyclopedia of Genes and Genomes orthologies and compared the performance of these orthology models to that of MGSs; (4) we assessed the robustness of microbiome-associated metabolites across populations, that is, the Israeli^14^ and British TwinsUK^23^ cohorts versus the Swedish cohort; (5) we verified whether the predicted microbiome-associated metabolites were significantly altered in germ-free (GF) versus conventionally raised (CONV-R) mice.

**Extended Data Fig. 2:**
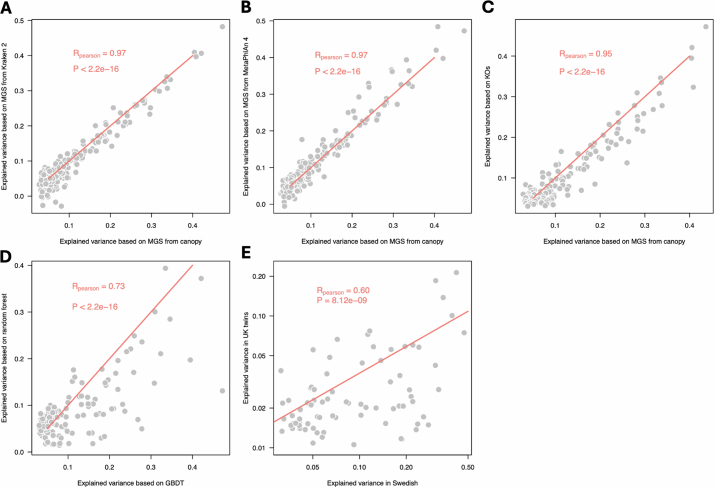
Robust predictions of gut-microbiome associated metabolites (n = 197). **A-C**. Explained variance in microbial metabolites attributed to metagenomic species (MGSs) as assessed by reference-free canopy clustering versus reference-based Kraken 2 (**A**), lineage-specific marker genes based MetaPhlAn 4 (**B**), and functional KEGG orthologies (**C**), respectively. **D**. Comparison of explained variance in microbial metabolites by the gut microbiome using GBDT versus random forest algorithms. **E**. Explained variance in microbial metabolites by the gut microbiome in the Swedish versus UK twins cohorts. Two-sided Pearson correlation analyses were performed with raw p values reported for results in A-E.

We observed that microbiome–metabolome associations were consistent across pipelines, with a Pearson correlation of 0.97 (*P* < 2.2 × 10^−16^) between Canopy and Kraken 2 (Extended Data Fig. 2a) and between Canopy and MetaPhlAn 4 (Extended Data Fig. 2b), microbiome configurations at MGSs and Kyoto Encyclopedia of Genes and Genomes orthology levels (Pearson correlation coefficient *R* = 0.95; *P* < 2.2 × 10^−16^; Extended Data Fig. 2c), as well as when testing different computational methods (*R* = 0.73; *P* < 2.2 × 10^−16^; Extended Data Fig. 2d), and between populations (*R* = 0.74 and *P* < 2.2 × 10^−16^ in the Israeli^14^ versus Swedish cohorts; Fig. 2c). Robust microbiome–metabolome associations were also replicated in the British TwinsUK cohort^23^ (*R* = 0.60; *P* = 8.12 × 10^−9^; Extended Data Fig. 2e), despite the gap of 0.9 ± 1.3 years between the collection of fecal and blood samples.

**Extended Data Fig. 3:**
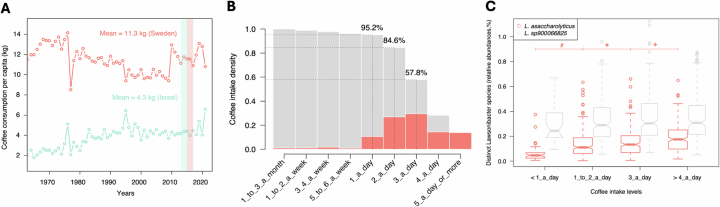
Coffee intake differences in Israeli versus Swedish. **A**. Trends in per capita coffee consumption (kg) in Sweden and Israel over the past 50 years (data source: Food and Agriculture Organization of the United Nations). **B**. Histograms in brown showing the raw coffee intake densities while that in grey showing the total densities over a specific coffee intake levels (n = 689). For instance, 95.2%, 84.6, and 57.8% individuals in this cohort took at least one, two, and three cups of coffee per day. **C**. Boxplots showing the relative abundance changes of distinct Lawsonibacter species (including the top two most abundant *L. asaccharolyticus* and *L. sp900066825*) along increased coffee intake levels (n = 689). Boxes show median (line), 25th/75th percentiles (box), and 1.5x the interquartile range (whiskers). Wilcox rank-sum test (two-sided); *, P = 0.0468( < 0.05); *, P = 0.00669( < 0.01);#, P = 7.045e-06( < 0.001).

Apart from the generally consistent microbiome–metabolome associations across populations, we identified 15 metabolites differently predicted by the microbiome between the Israeli and Swedish cohorts (Fig. 2c). These metabolites were dominated by xenobiotics, including 11 metabolites involved in benzoate (3-phenylpropionate) and xanthine metabolism (for example, caffeine and 5-acetylamino-6-amino-3-methyluracil), with the remaining four from amino acid metabolism (phenol sulfate, indole-acetate, phenylacetylglutamine and *p*-cresol-glucuronide). Interestingly, the eight xanthine-related metabolites involved in caffeine metabolism, along with quinate (a compound commonly found in coffee), were associated with diet in the Israeli cohort but not in Swedish cohorts. This may be attributed to distinct dietary habits. Epidemiological data and food logs show that coffee intake in the Israeli cohort is about one-third of that in the Swedish cohort, despite doubling over the past 50 years (data from the Food and Agriculture Organization of the United Nations^32^; Extended Data Fig. 3a). In agreement, 95.2% of individuals in the Swedish cohorts reported at least one cup of coffee per day, while 84.6% and 57.8% reported two or more than three cups of coffee per day, respectively (Extended Data Fig. 3b). Thus, we hypothesized that the gut microbiome of Swedes has adapted to routinely coffee exposure, and that the high intake of coffee may reduce the variability of these metabolites that can be attributed to diet. The relative abundances of *Lawsonibacter asaccharolyticus*, a bacterium involved in coffee metabolism^19,33^, were indeed lower in the Israeli cohort compared to the Swedish cohort (Fig. 2d). Furthermore, the abundance of this bacterium, but not other *Lawsonibacter* species including *Lawsonibacter sp900066825*, were associated with more frequent coffee consumption (Extended Data Fig. 3c).

GF and CONV-R mice offer a robust model to validate in-silico-predicted microbiome-associated metabolites in vivo. Thus, we performed metabolomic profiling of plasma from the portal vein of these mice and identified 66 of 197 microbiome-associated metabolites found in humans, with over half (54.5%) showing significant differences between the two models (Supplementary Table 6 and Fig. 2e), thus confirming their strong association with the gut microbiota.

Finally, we explored whether metabolites were associated with microbial diversity by assessing the Shannon index. Consistent with previous results^24^, we confirmed that interindividual differences in the gut microbiome were reflected in the plasma metabolome, explaining 49.4% of the variance in alpha diversity, whereas clinical biomarkers explained only 9.4% of the variance (Fig. 2f). Unexpectedly, we also observed that the explained variances by lipid-derived (39.8%) and amino-acid-derived (35.1%) metabolites were minimally additive (43.5% when combined), suggesting that the microbiome–lipid interactions were interconnected, either directly or indirectly, with microbiome–amino acid interactions^34,35^.

### Molecular signatures of individuals with impaired glucose control

**Fig. 3:**
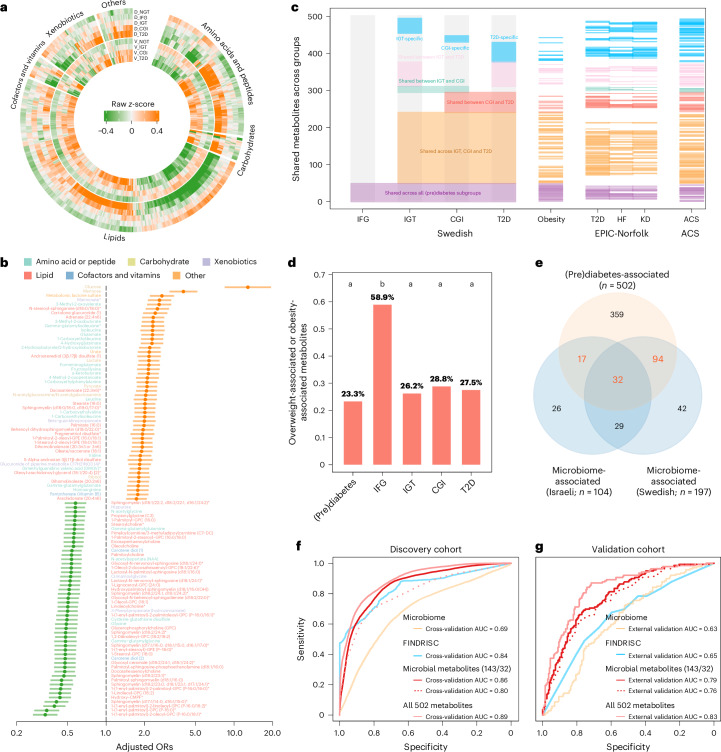
Molecular signatures of distinct subgroups with impaired glucose control and comorbidities. **a**, Circular heatmap showing the 502 metabolites consistently altered in the prediabetes and T2D groups versus the NGT group in both the discovery (D) and validation (V) cohorts (Wilcoxon rank-sum test; two-sided *P*_adj_ < 0.1). **b**, Top 100 metabolites with significantly lower or higher ORs of CGI/T2D risks after adjusting for age and sex (logistic regression analyses; two-sided *P*_adj_ < 0.1) **c**, Metabolites uniquely associated with specific prediabetes and T2D subgroups and those shared across groups. Metabolites associated with overweight or obesity (*n* = 117 of 165) in the NGT group of the discovery cohort, T2D (*n* = 150), HF (*n* = 99) and KD (*n* = 111) in the EPIC-Norfolk cohort, and ACS (*n* = 205) in Israelis are highlighted with colored lines if they were also T2D-associated in Swedes. **d**, Proportions of overweight-associated and obesity-associated metabolites over all prediabetes-associated, IFG-associated, IGT-associated, CGI-associated or T2D-associated metabolites. Groups labeled with different letters (a or b) indicate significant statistical differences (two-sided chi-squared test). **e**, Venn diagram showing that a total of 143 microbiome-associated metabolites (calculated by summing the three numbers highlighted in red) identified in Israelis (*n* = 104) or Swedes (*n* = 197) overlap with prediabetes-associated and T2D -associated metabolites (*n* = 502). **f**,**g**, Random forest classifiers in distinguishing CGIs and T2Ds from NGTs in the discovery (**f**) and validation (**g**) cohorts based on the FINDRISC, microbiome, 143 microbiome-associated and prediabetes-associated and diabetes-associated metabolites, the 32 most robust microbiome-associated metabolites identified in both the Swedish and Israeli cohorts, or all 501 of 502 prediabetes-associated and diabetes-associated metabolites excluding glucose. The performance of the classifiers is assessed by AUC; the cross-validation AUCs based on tenfold cross-validation repeated ten times in the discovery cohort and true prediction AUCs in the validation cohort were provided, respectively.

In total, we identified 64, 510, 450 and 585 metabolites that showed significantly altered plasma levels in individuals with isolated IFG, isolated IGT, CGI and T2D, respectively, compared to the NGT controls in the discovery cohort (Wilcoxon rank-sum test, *P*_adj_ < 0.1), resulting in 759 potential metabolites associated with impaired glucose control (Supplementary Table 7). Of these molecular signatures, 502 were altered in the validation cohort, of which 54.2% were annotated as lipid-related and 20.3% as amino-acid-related metabolites (Fig. 3a and Supplementary Table 7). However, imidazole propionate, one of the top ranked microbiome-associated metabolites (Fig. 2e), was only significantly increased in the IGT cohort in individuals with impaired glucose control versus NGT (discovery) but not in SCAPIS (validation, *P*_adj_ = 0.11) cohort (Supplementary Table 7); accordingly, it was not included in the downstream analyses. Of the 502 metabolites, 469 (126 microbiome-associated), remained significantly associated with higher and lower odds ratios (ORs) for IFG, IGT or CGI/T2D, after adjusting for group differences in age and sex similarly to a previous study^9^ (Fig. 3b and Supplementary Table 8; logistic regression analyses; *P*_adj_ < 0.1).

Comparison of the altered metabolites across distinct prediabetes and T2D groups revealed that 56 of 502 metabolites were significantly altered in isolated IFG compared to the NGT control. Interestingly, these 56 metabolites were concurrently altered in all subgroups of prediabetes and T2D, prompting the question of whether, and to what extent, IFG and IGT are fundamentally different (Fig. 3c and Supplementary Table 7). In contrast, 241 (48.0%) of altered metabolites were shared among subgroups characterized by glucose intolerance (isolated IGT, CGI and T2D).

Metabolites linked to prediabetes or diabetes were then compared with those associated with distinct cardiometabolic diseases to identify potential shared metabolic pathways between the two conditions^9,13^. When the 220 individuals with NGT in the discovery cohort were stratified according to BMI, 165 metabolites were significantly altered in overweight or obese individuals (BMI ≥ 25; *n* = 108) compared to the lean controls (BMI < 25; *n* = 112) (Fig. 3c and Supplementary Table 7). Of these, 117 (70.9%) overlapped with the 502 prediabetes-associated and T2D-associated metabolites but only eight were identified as overweight-specific and obesity-specific (including malonate, methylmalonate, cortisol, myo-inositol, 2-palmitoylglycerol (16:0), 2-linoleoylglycerol (18:2), 3β-7α-dihydroxy-5-cholestenoate and N-δ-acetylornithine) (Supplementary Tables 7 and 9). A connection between cortisol and obesity is well established^36^ and gut bacteria metabolizing myo-inositol were recently suggested to be enriched in an obesity-related gut microbiome enterotype^37^. In addition, 33 obesity-associated metabolites were identified in the isolated IFG group, accounting for 58.9% of altered metabolites in this subgroup, which constituted a significantly larger proportion than in isolated IGT (26.2%), CGI (28.8%) or T2D (27.5%) groups (chi-squared test, *P* < 0.01) (Fig. 3d). Our results also demonstrated that 245 of 502 metabolites were associated with noncommunicable diseases in the EPIC-Norfolk cohort^9^, which included 150 associated with incidence of T2D, 99 with HF and 111 with kidney disease (KD) (Fig. 3c and Supplementary Table 9). We also observed that 392 of 533 metabolites showing altered differences between ACS and non-ACS controls^11^ were detected in our study. Notably, 52.3% (205 of 392) were consistently associated with prediabetes and T2D (Fig. 3c and Supplementary Table 9), which is not unexpected as 31.2% of patients with ACS had T2D^11^. This conclusion is supported by studies indicating that similar microbiome and metabolome alterations are observed across the span of cardiometabolic diseases from obesity to HF^12^.

**Extended Data Fig. 4:**
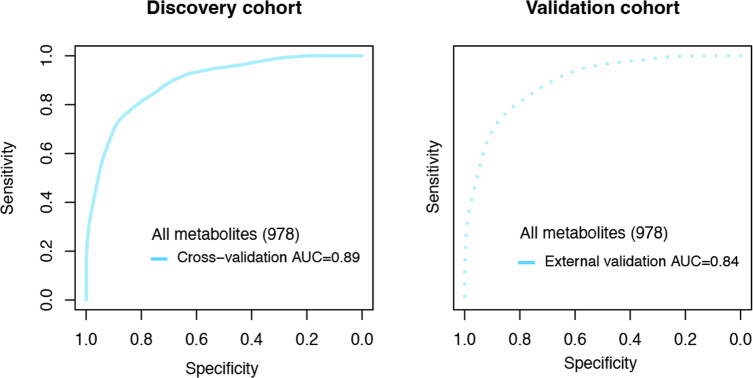
Random Forest classifier in distinguishing CGIs/T2Ds from NGTs based on all metabolites. The performance of the classifiers is assessed by area under the curve (AUC); the cross-validation AUCs based on 10-fold cross-validated repeated 10 times in the discovery cohort and external prediction AUCs in the validation cohort were provided, respectively.

Among the 502 metabolites identified as potential biomarkers of impaired glucose control, 143 were microbiome-associated in either the Swedish or Israeli cohort (Fig. 3e). We performed random forest classification to compare the metabolome’s ability to distinguish CGI and T2D from NGT controls, versus microbiome-based classifiers and FINnish Diabetes Risk SCore (FINDRISC), which showed similar performance^25^. The models were trained and optimized in the discovery cohort, then applied to the validation cohort for prediction. Model performance was assessed using the area under the curve (AUC). The non-glucose-metabolite-based (*n* = 501) classifiers demonstrated superior performance compared to their MGSs classifiers and the FINDRISC score, with AUCs of 0.89 and 0.83 in the discovery and validation cohorts, respectively (Fig. 3f), comparable to models using all metabolites without preselection (AUCs of 0.89 and 0.84, respectively; Extended Data Fig. 4). Models based on the 143 microbiome-associated metabolites linked to impaired glucose control, or the 32 metabolites robustly associated with the gut microbiome across populations, also showed superior performance compared to the MGSs classifier, with AUCs of 0.79 and 0.76, respectively, in the validation cohort (Fig. 3g).

### Diet–microbiota interactions affecting glucose control

We next conducted feature attribution analysis based on the SHapley Additive exPlanation (SHAP) approach to identify the potential effects of specific MGSs and lifestyle factors on plasma molecular signatures affecting glucose control. SHAP values quantify feature importance and attribute gut microbiome taxa contributions to functional perturbations while preserving microbial composition^38^. We focused on the 502 consistently changed metabolites in the two Swedish cohorts and 118 MGSs associated with prediabetes and T2D in the same individuals identified previously^25^ (Supplementary Table 3).

**Extended Data Fig. 5:**
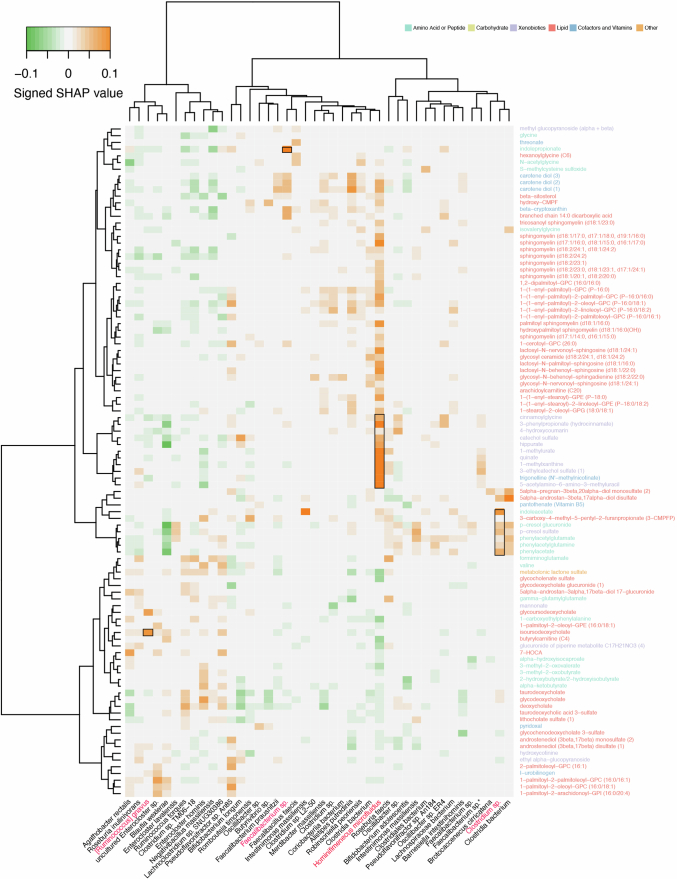
Subset of a heatmap showing the top metabolites-MGS pairs. The heatmap showing the top most important metabolites-MGS (metagenomic species) pairs based on signed SHAP values. Key pairs highlighted in black boxes.

**Extended Data Fig. 6:**
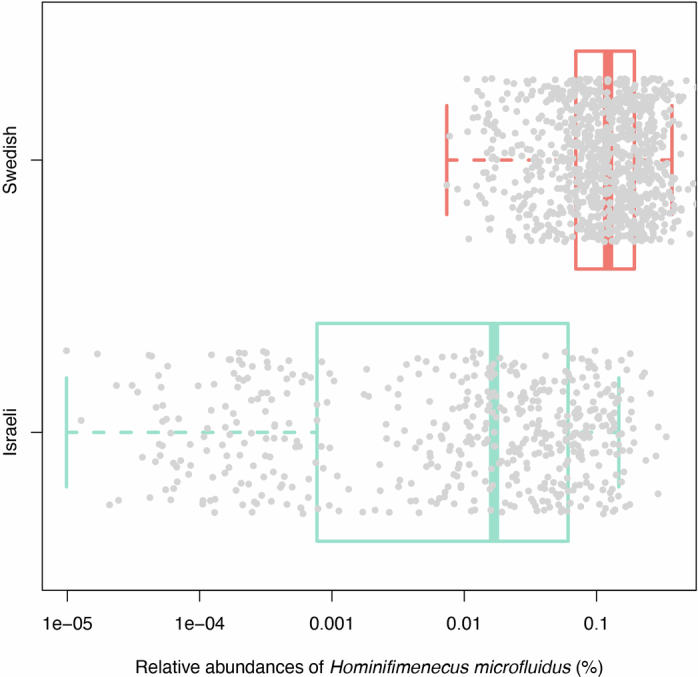
The relative abundances *Hominifimenecus microfluidus* in Israeli versus Swedish. Boxplots showing the distinct relative abundances of *H. microfluidus* (%) in Israeli and Swedish cohorts, respectively. The sample sizes are 969 and 1,167, respectively. Boxes show median (line), 25th/75th percentiles (box), and 1.5x the interquartile range (whiskers).

Our results indicate that among the MGS–metabolite pairs with the highest SHAP values, the recently isolated but largely uncharacterized species *Hominifimenecus microfluidus* can have a significant impact on the metabolism of several xenobiotics, including quinate (Extended Data Fig. 5 and Supplementary Table 10). As expected, the variation in abundance of this bacterium is similar to *L. asaccharolyticus* (Fig. 2d), exhibiting much lower abundances in Israelis compared to Swedes (Extended Data Fig. 6). *Faecalibacterium* species are among the key features associated with indolepropionate levels, which are inversely associated with the risk of T2D, consistent with previous findings^39^. Another notable MGS–metabolite pair was observed between *Ruminococcus gnavus* and isoursodeoxycholate, which is consistent with the known ability of *R. gnavus* to produce iso-bile acids^40^. The capacity to produce isoursodeoxycholate may provide a mechanism for how *R. gnavus* contributes to inflammation and cardiometabolic disease^41^. Additionally, predicted plasma levels of metabolites involved in phenylalanine metabolism, such as phenylacetate, phenylacetylglutamate and phenylacetylglutamine, were linked with certain bacteria of the *Clostridium* genus, which are linked to heightened cardiovascular disease risk^42,43^.

**Fig. 4:**
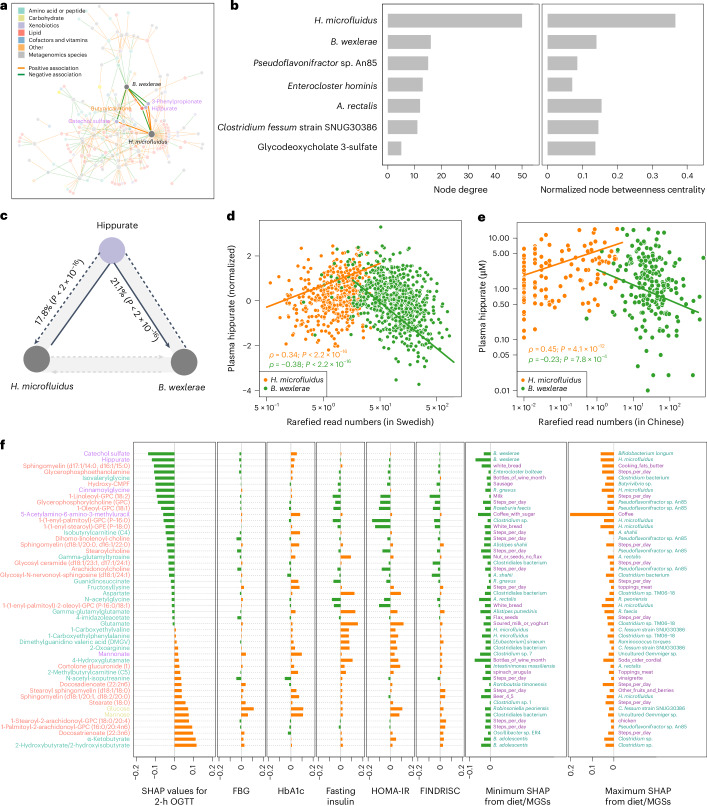
Gut microbial features explaining glucose intolerance. **a**, Bi-network showing the top 300 MGS–metabolite pairs with the largest absolute SHAP values based on a force-directed algorithm. **b**, Network analysis of the node degree and betweenness centrality for those top 300 MGS–metabolite pairs. **c**, Bidirectional causal inference using mediation analyses to estimate the proportions of effect mediated by hippurate between *H. microfluidus* and *B. wexlerae*. **d**, Dot plot showing the significant correlations between plasma hippurate and *H. microfluidus* and *B. wexlerae*, respectively, in the discovery cohort (Spearman *ρ* correlation analyses, raw two-sided *P* < 2.2 × 10^−16^). **e**, Replication of the *Hominifimenecus*-hippurate-*Blautia* associations in a Chinese cohort (Spearman *ρ* correlation analyses, two-sided raw *P* < 2.2 × 10^−16^). **f**, Top ten metabolites identified as important features in the 2-h OGTT, FBG HbA1c, fasting insulin, HOMA-IR or FINDRISC (*n* = 49 in total) based on the GBDT models; metabolites were order according to their SHAP values (reflecting feature importance) to 2-h OGTT levels. The lifestyle features (purple) including both the physical activity levels (as measured by steps per day) and dietary components or MGSs (green) with the maximum or minimum SHAP values for each metabolite are also shown on the right. Metabolites were colored using pathway annotations, including those involved in amino acid, lipid, carbohydrate and xenobiotic metabolism as in **a**.

To gain a broader understanding of the interactions between the plasma metabolome and different predictive MGSs, we then used the top 300 metabolite–MGS pairs with the strongest SHAP values for dynamic network visualization using a force-directed algorithm. Notably, this analysis identified *H. microfluidus* and *Blautia wexlerae*, both members of the Lachnospiraceae family, as the key nodes of the metabolome–microbiome dynamics in prediabetes and T2D (Fig. 4a). Further network analysis confirmed these observations: three MGSs—*H. microfluidus*, *B. wexlerae* and *Agathobacter rectalis*—were consistently ranked among the top five features based on their high node degree and betweenness centrality, potentially acting as keystone species (Fig. 4b). Interestingly, we observed an inverse relationship between *H. microfluidus* and *B. wexlerae* via four metabolites, of which three were involved in benzoate metabolism, including catechol sulfate, 3-phenylpropionate and hippurate (Fig. 4a,b). The tight connections between hippurate and different *Blautia* species and strains have also been observed in the LifeLines DEEP cohort^16,44^. Additional mediation analyses revealed a bidirectional relationship between these two bacteria: hippurate mediates 21.1% of the effect of *H. microfluidus* on *B. wexlerae*, while 17.8% of the effect of *B. wexlerae* on *H. microfluidus* is mediated through this metabolite (Fig. 4c). Note that the SHAP-based analyses were consistent with Spearman correlation analyses both in the Swedish cohort (Fig. 4d) and in a geographically independent Chinese cohort where we had previously profiled the gut microbiome using the same methods^45^ (Fig. 4e). These findings are consistent with a gut microbiome structure consisting of two competing guilds across population and health status^46^.

**Extended Data Fig. 7:**
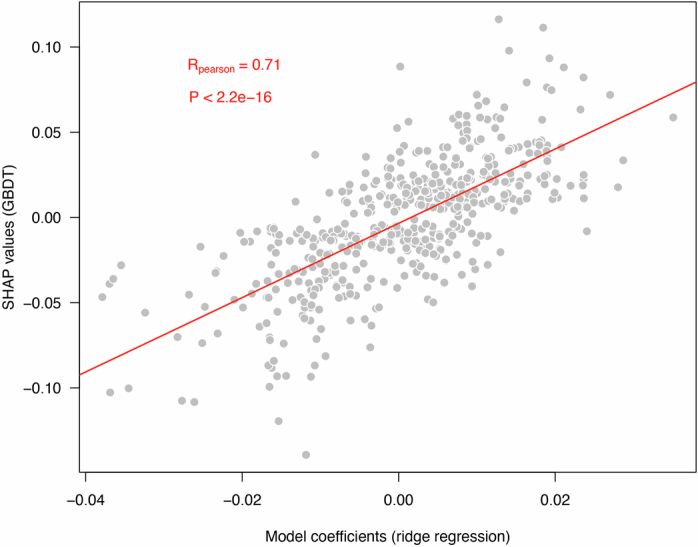
Robust correlation between SHAP values and linear regression coefficients. The SHAP values of metabolites in relation to the 2-hour OGTT levels derived from GBDT and the corresponding model coefficients for each metabolite based on linear ridge regression analyses were compared using two-sided Pearson correlation analysis.

Next, we assessed the SHAP values of metabolites in relation to several glucose and insulin indices in our cohorts, encompassing 2-h OGTT levels, fasting blood glucose (FBG), hemoglobin A1c (HbA1c), fasting insulin, homeostatic model assessment of insulin resistance (HOMA-IR) and FINDRISC (Fig. 4f and Supplementary Table 11). Our findings revealed that the primary metabolites reflective of FINDRISC were generally consistent with those influenced by fasting insulin and HOMA-IR but not FBG, suggesting that FINDRISC may reflect insulin resistance rather than glycemia per se from a molecular perspective. Of interest, catechol sulfate and hippurate emerged as the top two features exhibiting negative contributions with 2-h OGTT levels, but not with fasting insulin and HOMA-IR, to which the most positive and negative contributions were glutamate and 1-(1-enyl-palmitoyl)-GPC (*P*-16:0), respectively. Our results further indicated that *Bifidobacterium adolescentis* was linked to lower levels of α-ketobutyrate and 2-hydroxybutyrate, which showed the highest positive SHAP values with 2-h OGTT (Fig. 4f). Note that the SHAP values of metabolites regarding the 2-h OGTT strongly correlated with the model coefficients from the linear ridge regressions, demonstrating robustness across distinct ML methods (*R* = 0.71, *P* < 2.2 × 10^−16^; Extended Data Fig. 7).

### Lifestyle-specific modulation of diabetes-linked metabolites

In line with the established understanding that both gut microbiota and T2D are influenced by lifestyle changes^10,22^, our analyses identified physical activity levels (measured as steps per day; Fig. 4d) and several dietary components to be among the top factors influencing variations in distinct diabetes-related metabolites. Thus, we analyzed the plasma metabolome data from two previous longitudinal trials—one focusing on diet^22^ and the other on exercise^47^—to identify molecular responses to these lifestyle interventions. We could determine the levels of 307 of 502 metabolites associated with impaired glucose control; of these, 125 were associated with improvement in insulin sensitivity (as reflected by HOMA-IR) upon dietary intervention^22^. Most of these metabolites were lipids (77, 61.6%), amino acids (39, 31.2%) and xenobiotics (9, 7.2%) (Supplementary Table 12);123 of 125 metabolites overlapped with the metabolites profiled in the exercise intervention study aiming to characterize the metabolic benefits of short-term exercise^47^ (Supplementary Table 12).

**Fig. 5:**
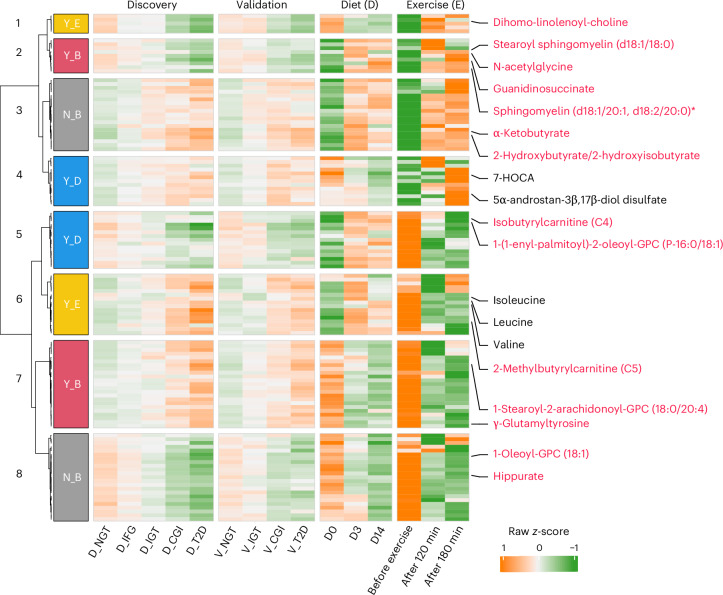
Responses of prediabetes and T2D-associated metabolites to a 2-week diet intervention or before and after exercise. Heatmap showing the overlapping metabolites involved in amino acid, lipid and xenobiotic metabolism (*n* = 123) in two clinical trials of either diet (14 days) or exercise for 1-h (before, 120 and 180 min after exercise) interventions with those 502 altered metabolites in prediabetes and T2D. Responses reversed (Y, yes; N, no) by either diet (D) or exercise (E) or both (B) were clustered and are shown in distinct colors beside the row clustering branches. Representative metabolites including 14 overlapping with Fig. 4f are labeled in red, and five others in black. Wilcoxon rank-sum test and one-way repeated-measures analysis of variance were used to identify altered metabolites in the cohorts and two longitudinal datasets (*P*_adj_ < 0.1), respectively.

Additional hierarchical clustering analysis revealed that the 123 overlapping metabolites could be classified into eight clusters based on their differences in prediabetes and T2D versus NGTs, and their responses to both the dietary and exercise interventions (Fig. 5). Eighty-one (65.9%) of these metabolites responded to at least one of the interventions. Importantly, we observed that lifestyle–metabolite interactions varied depending on the type of intervention (Fig. 5 and Supplementary Table 12), similar to the heterogeneity observed in T2D pathogenesis. Specifically, 32 metabolites were reversible after both interventions (clusters 2 and 7), while 42 metabolites were not altered by either intervention (clusters 3 and 8). Moreover, 28 metabolites showed reversal only after the dietary intervention (clusters 4 and 5), whereas 21 metabolites responded exclusively to exercise (clusters 1 and 6).

**Extended Data Fig. 8:**
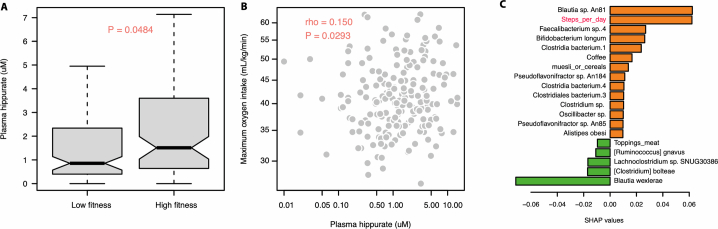
Factors modulating circulating hippurate levels. **A**. Plasma levels of hippurate in individuals with low versus high physical fitness levels in a Chinese athlete cohort (n = 213, two-sided Wilcox rank-sum test). Boxes show median (line), 25th/75th percentiles (box), and 1.5x the interquartile range (whiskers). **B**. Spearman correlation between plasma hippurate levels and maximum oxygen intake levels in this Chinese cohort (two-sided). **C**. Top gut metagenomics species and lifestyle components displayed the most associations (absolute SHAP values ≥ 0.1) with circulating hippurate levels in the Swedish IGT cohort.

Interestingly, 14 of the top 49 features associated with glucose or insulin indices (Fig. 4d) were also identified and ten of these were reversible using short-term lifestyle changes; the remaining four—hippurate, 1-oleoyl-GPC (18:1), α-ketobutyrate and 2-hydroxy(iso)butyrate (Fig. 5)—were not. This indicates that other factors modulate these metabolites. In support, significantly elevated plasma hippurate levels were observed in the Chinese cohort when stratified according to high versus low physical fitness levels (*P* = 0.048; Extended Data Fig. 8a) and correlated with maximum oxygen intake levels (Extended Data Fig. 8b), suggesting that long-term, but not short-term, physical exercise might modulate this microbial metabolite. In agreement, average daily steps, which are indicative of habitual physical activity, emerged as the second most influential factor positively associated with circulating hippurate levels in our Swedish cohort (Extended Data Fig. 8c). The plasma levels of three branched-chain fatty acids, repeatedly linked to glucose control and insulin resistance^48^, could be reduced with short-term exercise but, as expected, not with a high-protein diet. In contrast, 7α-hydroxy-3-oxo-4-cholestenoic acid (7-HOCA), a new substrate of liver 5β-reductase contributing to liver lipid dysregulation^49^, and 5-α-androstan-3-β,17-β-diol disulfate, a top feature associated with alcohol consumption^50^, could only be reduced by diet and not exercise intervention. Thus, our results indicated that the interactions between lifestyle and the microbiome–metabolome axis are modifiable targets for T2D management; however, optimal health benefits might be achievable through a combination of lifestyle modifications.

## Discussion

We identified 502 metabolites linked to altered glucose control in treatment-naive individuals, with 143 correlating to the gut microbiome, suggesting that microbiome–metabolome disruptions contribute to glucose control changes. Validation in external cohorts highlighted a subset of microbiome-associated metabolites in Swedes and Israelis that effectively identify prediabetes and T2D. We identified a series of molecular signatures that could serve as biomarkers or therapeutic targets for evaluating the impact of lifestyle on metabolic health through gut microbial modulation.

IFG and IGT are two prediabetes subgroups reflecting hepatic and peripheral insulin resistance, respectively^51,52^. Our metabolomics analysis revealed both shared and IGT-specific metabolite alterations, aligning with IGT’s stronger association with environmental factors like physical inactivity and unhealthy diets^52^. In support, we previously observed more pronounced microbiome alterations in individuals with IGT compared to those with IFG^25^. Additionally, our metabolomic profiling revealed significant overlap in molecular signatures between prediabetes, T2D and cardiometabolic diseases like ACS^11^ and HF^9,13^. This confirms that the microbiome–metabolome axis is altered long before the development of cardiovascular disease^12^, highlighting the need for early interventions targeting the gut microbiome in cardiometabolic diseases.

The data integration analyses further enabled us to characterize the prediabetes-associated and T2D-associated metabolites and their potential predictive MGS and diet factors. A total of 502 metabolites, including 143 of microbial origin, were identified as molecular signatures of prediabetes and T2D, particularly those involved in benzoate metabolism, such as hippurate by *H. microfluidus*. In agreement, recent research identified hippurate as a key metabolite linked to gut microbial diversity and negatively associated with metabolic syndrome in the TwinsUK cohort^53^. Moreover, hippurate has been proposed as a mediator of metabolic health, improving glucose tolerance in obese mice^54^. Mechanistically, it may act by reducing circulating urate^55^, a risk factor for T2D^56^ and atherosclerosis^57^. Overall, our data suggest that microbiome-associated metabolites, alone or with host-specific ones, have greater potential as biomarkers for prediabetes and T2D compared to FINDRISC and the microbiome itself, despite their complexity.

Another key aspect of our study is the recognition that heterogeneity exists not only in T2D development but also in corresponding interventions^6^. Lifestyle modifications are well known for reducing T2D incidence^58^, but their potential impact on the blood metabolome has not been thoroughly investigated. We demonstrated that approximately 65.9% of metabolites associated with T2D might be reversible by specific diet or exercise interventions. However, optimal health benefits arise from combining lifestyle modifications, as their physiological effects and clinical outcomes vary from a molecular perspective. This supports the idea that adding exercise to calorie restriction improves beta cell function in patients newly diagnosed with T2D^59^.

Our study has inherent limitations associated with the cross-sectional study design^25^; we also only performed metabolomic analyses on fasting samples at a single time point. However, key findings were validated in both geographically dependent and independent cohorts; the distinct metabolic responses to short-term diet and exercise interventions, either transient or extended, in two clinical trials, partly supports the molecular signatures defined for prediabetes and T2D. Moreover, to analyze a broad panel of metabolites, we performed semi-quantitative (qualitative) analyses and thus quantitative (for example, stable isotope dilution) liquid chromatography–tandem mass spectrometry (LC–MS/MS)-based assays may improve the clinical association and capture greater variation in prediabetes-related and T2D-related clinical phenotypes. Finally, longitudinal follow-up studies of prediabetes are required to determine the potential of using these metabolites to predict disease development.

In summary, our examination of microbiome–metabolome interactions identified over 500 metabolites associated with varying degrees of glucose control, with one-third linked to the gut microbiota. Understanding the connections between diet, gut microbiota and clinical factors provides valuable insights into T2D and highlights the need for diverse intervention strategies. This resource may provide increased understanding for how the gut microbiota may affect T2D and help identify new targets for diabetes management.

## Methods

### Description of cohorts

#### IGT and SCAPIS cohorts

We analyzed plasma samples and banked data from two previously collected Swedish prediabetes cohorts (aged 50–64 years): the IGT (discovery) and a subset of the SCAPIS (validation) cohorts^25^, for a total of 1,167 samples. The study was approved by the Ethics Review Board in Gothenburg (nos. 560-13, 2010-228-31M and 2013-365-32M); all participants gave informed written consent. The study design, including the inclusion and exclusion criteria and glucose status, have been described elsewhere^25,60^. Briefly, participants were invited to a fasting capillary blood glucose measurement and a 75-g OGTT in the morning between 7:30 and 11:00, when a fasting venous blood sample was also collected. Participants were then stratified into different subgroups, including those with NGT, isolated IFG, isolated IGT, CGI and newly diagnosed T2D, according to their fasting and 2-h OGTT capillary plasma glucose levels using the 1999 World Health Organization criteria^61^.

Fecal samples were collected at home and stored at room temperature for a maximum of 36 h before delivering to −80 °C storage within 2 weeks upon glucose measurements. Participants also completed a detailed questionnaire based on questions used in previous epidemiological studies (https://www.scapis.se/) and the FINDRISC, a well-validated eight-item European questionnaire developed to identify individuals at high risk of future diabetes^62^. FINDRISC ranges from 2 to 25 and is calculated based on collected information about age, sex, weight and height, waist circumference, use of concomitant blood pressure medication, history of high blood glucose disorders, physical activity, family history of diabetes and diet^62^. The high-risk NGT group diagnosed based on FINDRISC (mean ± s.e.m. = 15.5 ± 0.6) without gut microbial changes (*n* = 297)^25^ and 31 samples without plasma from both cohorts were excluded from the metabolomics profiling and downstream analyses.

Clinical data, including basic anthropometric measurements, traditional systematic inflammation markers, and lipid, glucose and insulin indexes (Supplementary Table 1) were measured or calculated as described previously^25^. The dietary information for each individual based on MiniMeal-Q, a validated web-based interactive FFQ consisting of 45 food categories, 126 food items and 193 questions^28,29^ was collected (Supplementary Table 4). All FFQ responses were converted to numeric values based on the frequency of consumption, enabling statistical modeling and analysis. However, 26 questions were excluded from the regression models because of missing values in over 80% of participants (Supplementary Table 4).

#### Intervention cohorts

Ten individuals with both obesity and T2D (age = 54 ± 4 years; BMI = 32.1 ± 3.8 kg m^−^^2^ (mean ± s.e.m.)) were instructed to follow a low-carbohydrate, high-protein and high-unsaturated fatty acids diet for 14 days and came to the study center on days 0, 3 and 14 of the diet after an overnight fast to deliver the fecal samples when the fasted plasma samples were also collected^22^. Significant decreases in fasting insulin and HOMA-IR values, in addition to rapid liver fat reduction upon the diet intervention, were observed^22^. Another ten young, healthy males (age = 24 ± 1 years; BMI = 23.7 ± 0.6 kg m^−^^2^) were investigated before and after an acute exercise bouts for 1 h to study the health benefits of exercise. Participants were fasted overnight and exercise was performed in the morning when the blood samples were collected^47^. Plasma metabolome samples generated before and during a 3-h recovery phase at +120 and +180 min were used for analysis in this study.

#### Other cohorts

Metagenome data (*n* = 969) were analyzed, with microbiome-associated metabolites predicted based on 491 individuals with paired metabolome data from healthy Israeli individuals (aged 18–70 years), as described previously^14^. Metabolome data from individuals with ACS (*n* = 199; aged 30–80 years) were retrieved from Talmor-Barkan et al.^11^. Blood metabolomics for both cohorts were profiled using the Metabolon platform, but samples were not collected under strict fasting conditions. Notably, 31.2% of the individuals with ACS had diabetes comorbidity^11^. Fecal samples from the TwinsUK study were collected at home, refrigerated for up to 2 days and then stored at −80 °C at King’s College London. Blood samples were collected during clinical visits, averaging 0.9 ± 1.3 years apart from the fecal sample collection^23^. In total, 606 individuals with matched metabolome and microbiome data were analyzed from the TwinsUK study. Plasma samples from non-fasted individuals were collected and stored in liquid nitrogen before metabolomics profiling using the Metabolon platform in the EPIC-Norfolk study^9^. In the Chinese athlete cohort, fecal samples were collected and stored at −20 °C for up to 24 h before being transferred to −80 °C storage during their clinical visit, when overnight fasting plasma samples were also collected^45^ and subjected to metabolomics profiling as described previously^13^(*n* = 213 with matched fecal and blood samples).

### Fecal microbial profiling and analysis

The metagenomics study of individuals from the IGT and SCAPIS cohorts was reported in our previous study^25^. Briefly, an average of 26.5 million high-quality paired-end reads were generated after quality control and used for assembling 15,186,403 nonredundant gut microbial genes. Taxonomic annotations for these genes were performed using BLASTN^63^ against NCBI reference sequences (release 224)^64^. The updated gut microbial gene catalog and annotations are available at https://omicsdata.org/download/IGT_scapis_catalog/. Gene abundance profiles were generated by mapping reads to this gene catalog using Bowtie 2 (ref. ^65^) and were rarefied to 22 million reads per sample (mean across 50 repeated rarefactions) for all cohorts before the downstream analyses. In total, 1,427 co-abundant gene groups, known as MGSs, were identified based on the rarefied gene abundance table using the canopy clustering algorithm^66^ (Supplementary Table 3). Among these, 118 MGSs were consistently altered in prediabetes and T2D compared to the NGT groups (*P*_adj_ < 0.1)^25^; 19 MGSs were assigned new taxonomic names based on the updated NCBI RefSeq annotations (Supplementary Table 3).

Two complementary metagenomic pipelines, including the reference genome-based Kraken 2 software suite^30^ and the lineage-specific marker-gene-based MetaPhlAn 4 (ref. ^31^), were used to evaluate the robustness of the taxonomic profiles derived from the reference-free canopy clustering methods described above^25^. Briefly, raw sequencing reads were processed using Trimmomatic^67^ (v.0.39) for quality control and trimming. Human-derived reads were filtered out by aligning the remaining reads to the human genome reference (hg38) using Bowtie 2 (ref. ^65^). High-quality, nonhuman reads were retained for downstream taxonomic profiling. In the Kraken 2 pipeline, reads were aligned using a *k*-mer-based approach against the Unified Human Gastrointestinal Genome database (v.2.02)^68^. Bayesian re-estimation of abundance with Kraken^30^ was subsequently applied to generate species-level abundance profiles.

### Plasma metabolomics and preprocessing

Paired fasting plasma samples were subjected to metabolomics measurements based on the Metabolon platforms as described previously^69^. Briefly, samples were prepared using the automated Microlab STAR system (Hamilton Microlab). The extract after removal of proteins was divided into four fractions: two for analysis by two separate reverse-phase ultra-performance LC–MS/MS methods with positive ion mode electrospray ionization (ESI); one for analysis by reverse-phase ultra-performance LC–MS/MS with negative ion mode ESI; and one for analysis using hydrophilic interaction liquid chromatography/reverse-phase ultra-performance LC–MS/MS with negative ion mode ESI. ultra-performance LC–MS/MS analysis was based on the Waters ACQUITY ultra-performance LC and a Thermo Scientific Q-Exactive high resolution accurate mass spectrometer interfaced with a heated electrospray ionization (HESI-II) source and Orbitrap mass analyzer operated at 35,000 mass resolution. Raw data were then extracted, peak-identified and quality-control-processed using Metabolon’s informatics system.

A total of 978 annotated metabolites were obtained and preprocessed as described previously^14^. Briefly, metabolites with fewer than ten measurements across the IGT or SCAPIS cohort were first removed, log_10_-transformed, imputed with the minimum value for each metabolite for the corresponding missing values and finally standardized (subtracting the mean and dividing by the s.d.).

Ten 15-week-old male GF and CONV-R C57BL/6J mice were fed an autoclaved chow diet (5021 LabDiet) ad libitum with unlimited access to water (*n* = 5/6 per group) as described previously^69^. All animals were kept in individually ventilated cages (ISOcage N System, Tecniplast) under a strict 12-h light cycle (light from 7:00 to 19:00), 20 ± 1 °C temperature and air humidity of 45–70%. Portal vein blood samples were then collected, stored at our animal facility and approved by the Ethics Committee on Animal Care and Use in Gothenburg, Sweden. Further untargeted metabolomics profiling of the portal vein plasma samples (*n* = 548 analytes) was performed using the same platform by Metabolon.

### Data integration and identification of microbially associated metabolites

Two distinct ML methods, including gradient-boosting decision trees (LightGBM v.2.1.1)^70^ and random forest (caret v.6.0-88)^71^ models, were used for data integration, in particular to predict metabolite levels and to compute the coefficient of determination (*R*^2^) between the predicted and measured values using the clinical, nutritional or microbial analytes. For the gradient-boosting tree models, we also computed the 95% confidence intervals and *P* values using 1,000 iterations of bootstrapping together with fivefold cross-validation for each bootstrap iteration using the Wald test, as described previously (Extended Data Fig. 1)^14^. Metabolites significantly associated with the microbiome data based on the adjusted *P* values were regarded as the microbiome-associated metabolites and further replicated in the Israeli^14^ and UK twins^23^ cohorts. Note that glucose and cholesterol in the metabolomics dataset were excluded from these analyses because of their inclusion as clinical variables.

SHAP values, a useful framework that can be used to reflect feature importance in gut microbiome data^38^, were computed (TreeExplainer v.0.20.4)^72^ when performing gradient-boosting tree analyses to estimate the attributions of different features to each metabolite. The best models were chosen over a random hyperparameter search consisting of ten iterations for each cross-validation fold (scikit-learn v.0.20.4)^73^. The SHAP values calculated across the clinical, nutritional, metabolomic and microbial data were visualized interactively using unsupervised force-direct network with networkD3 (v.0.4)^74^. A force-directed network has the advantage of spatially grouping similar features from heterogeneous data^75^.

### Statistical analysis

All statistical analyses were performed in R^76^ except the gradient-boosting decision trees, which were developed using Python 2.7.8 as described above and previously^14^. Two-tailed Wilcoxon rank-sum tests and repeated-measures one-way analysis of variance were used for the case-control and longitudinal analyses, respectively. The Shapiro–Wilk normality test was used to assess whether the data followed a normal distribution. The strength and direction of monotonic relationships between two variables were assessed using the Spearman rank-order correlation unless significant collinearity was detected. In cases of strong collinearity, the Pearson product-moment correlation was computed.

Bidirectional mediation analysis was used to assess the mediating role of the metabolite in the relationship between MGS1 and MGS2. Two linear models were applied: (1) the mediator model, that is, metabolite = MGS1 + age + BMI + sex; (2) the outcome model, that is, MGS2 = MGS1 + metabolite + age + BMI + sex. Direct, indirect and total effects were estimated using the mediate function from the R package mediation (v.4.5.0)^77^. The proportion of the effect mediated by the metabolite was calculated as the ratio of the indirect effect to the total effect. Statistical significance and 95% confidence intervals were determined using 1,000 bootstrap iterations. Covariates were controlled for and collinearity was checked to ensure the robustness of the results. The OR for the risk of prediabetes or diabetes for each metabolite was calculated based on logistic regression adjusted for age and sex. The random forest models were developed using the caret package (v.6.0-88)^71^ under the same parameter setting as described before (ntree = 5,000, metric = ‘kappa’, metric = ‘kappa’ for classification and ‘RSME’ for the regression analyses, respectively)^25^. The optimal model was determined through tenfold cross-validation, repeated ten times, using an upsampling strategy to balance group sample sizes, and selecting the tree with the highest kappa value. Ridge regression adjusting for age and sex was performed using the glmnet package (v.4.1-2); cross-validation (cv.glmnet) was used to select the optimal lambda. Model performance was evaluated with test predictions and coefficients were extracted using the best lambda. *P*_adj_ values were calculated using the qvalue package (v.2.24.0) based on default settings^78^. *P*_adj_ < 0.1 was considered significant unless otherwise stated.

### Reporting summary

Further information on research design is available in the Nature Portfolio Reporting Summary linked to this article.

## Online content

Any methods, additional references, Nature Portfolio reporting summaries, source data, extended data, supplementary information, acknowledgements, peer review information; details of author contributions and competing interests; and statements of data and code availability are available at 10.1038/s41591-025-03642-6.

## Supplementary information

Reporting Summary

Supplementary Tables 1–12 Supplementary Table 1: Clinical characteristics of the discovery and validation cohorts. Values reported as mean ± s.e.m.*, *P* < 0.05; +, *P* < 0.01; #, *P* < 0.001 versus the normal glucose tolerance control group. Supplementary Table 2: The 978 metabolites reported in this study and the corresponding pathway annotations. Supplementary Table 3: The 1,427 metagenomic species (MGSs) and their latest taxonomic annotations are presented. Of these, 118 MGSs associated with prediabetes and T2D from our previous study are indicated here; 19 MGSs have been assigned new taxonomic names (highlighted in red). Supplementary Table 4: The detailed food frequency questionnaire (FFQ) used and the numeric format for the collected FFQ responses. Supplementary Table 5: Explained variance for each metabolites in the discovery cohort according to clinical phenotypes, microbiome and diet, respectively. Supplementary Table 6: Overlapping metabolites in conventionally raised versus germ-free mice. Supplementary Table 7: All metabolites that significantly changed in the IFG, IGT, CGI or T2D compared to the normal glucose tolerance control group in both the discovery and validation cohorts. An additional 165 metabolites significantly altered in individuals with overweight or obesity (BMI ≥ 25) versus those with normal BMI (BMI < 25) in the normal glucose tolerance group of the discovery cohort are also labeled. Supplementary Table 8: Metabolites of lower and higher odds ratio for prediabetes or diabetes development in the entire Swedish cohort. Supplementary Table 9: Overlapping metabolites indicative of overweight or obesity in the discovery cohort, as well as in T2D, heart failure and kidney disease from the EPIC-Norfolk project, and acute coronary syndrome in the Israeli cohort. Supplementary Table 10: Metagenomics species or lifestyle components with maximum or minimum SHAP values for each of all 502 prediabetes and T2D associated metabolites. Supplementary Table 11: SHAP values of common glucose intolerance indices to each metabolite. Supplementary Table 12: Responses to diet and/or exercise for all 139 overlapping metabolites linked with the prediabetes and T2D across cohorts.

## Data Availability

The microbiome datasets are available at the Genome Sequence Archive for Human (accession no. HRA000020 at https://ngdc.cncb.ac.cn/gsa-human/browse/HRA000020 and accession no. HRA000933 at https://ngdc.cncb.ac.cn/gsa-human/browse/HRA000933, respectively). The Unified Human Gastrointestinal Genome catalog (v.2.02) is available at http://ftp.ebi.ac.uk/pub/databases/metagenomics/mgnify_genomes/human-gut/v2.0.2/. All summary statistics for the metabolome data for academic use can be accessed through an interactive web server (https://omicsdata.org/Apps/IGT_metabolome). The phenotypic data for the discovery cohort can be requested by contacting at fredrik@wlab.gu.se and from SCAPIS for the validation cohort according to the standard protocol for data access specified in detail at https://www.scapis.org/data-access/.

## Code availability

The Python code for the GBDT models is available at https://github.com/noambar/SerumMetabolomePredictions (ref. ^14^).
