# Supplementary material for: Microbiome–metabolome dynamics associated with impaired glucose control and responses to lifestyle changes
Source: Nat Med. 2025 Apr 8;31(7):2222–31. doi: 10.1038/s41591-025-03642-6 (PMC12283354; doi:10.1038/s41591-025-03642-6)
Supplement: Supplementary file 1 — Reporting Summary [file 41591_2025_3642_MOESM1_ESM.pdf]

nature portfolio

Corresponding author(s): Min Yue

Last updated by author(s): Mar 2, 2025

Reporting Summary

Nature Portfolio wishes to improve the reproducibility of the work that we publish. This form provides structure for consistency and transparency in reporting. For further information on Nature Portfolio policies, see our Editorial Policies and the Editorial Policy Checklist.

Statistics

For all statistical analyses, confirm that the following items are present in the figure legend, table legend, main text, or Methods section.

n/a	Confirmed	
☐	☒	The exact sample size (*n*) for each experimental group/condition, given as a discrete number and unit of measurement
☐	☒	A statement on whether measurements were taken from distinct samples or whether the same sample was measured repeatedly
☐	☒	The statistical test(s) used AND whether they are one- or two-sided<br>*Only common tests should be described solely by name; describe more complex techniques in the Methods section.*
☐	☒	A description of all covariates tested
☐	☒	A description of any assumptions or corrections, such as tests of normality and adjustment for multiple comparisons
☐	☒	A full description of the statistical parameters including central tendency (e.g. means) or other basic estimates (e.g. regression coefficient) AND variation (e.g. standard deviation) or associated estimates of uncertainty (e.g. confidence intervals)
☐	☒	For null hypothesis testing, the test statistic (e.g. *F*, *t*, *r*) with confidence intervals, effect sizes, degrees of freedom and *P* value noted<br>*Give P values as exact values whenever suitable.*
☐	☒	For Bayesian analysis, information on the choice of priors and Markov chain Monte Carlo settings
☐	☒	For hierarchical and complex designs, identification of the appropriate level for tests and full reporting of outcomes
☒	☐	Estimates of effect sizes (e.g. Cohen's *d*, Pearson's *r*), indicating how they were calculated

Our web collection on statistics for biologists contains articles on many of the points above.

Software and code

Policy information about availability of computer code

Data collection	No software was used for data collection.

Data analysis

Abricate v.1.0.1 (https://github.com/tseemann/abricate),
Snippy v.4.4.4 (https://github.com/tseemann/snippy),
SISTR v.1.1.1 (https://github.com/phac-nml/sistr_cmd),
Seqsero2 v.1.2.1 (https://github.com/denglab/SeqSero2)
SNP-dists v.0.7.0 (https://github.com/tseemann/snp-dists)
IQtree v.1.6.12 (https://github.com/iqtree/iqtree2).
Staramr v.0.9.1 (https://github.com/phac-nml/staramr)
Invasive Index (no version number)(http://www.github.com/UCanCompBio/invasive_salmonella)
Rhierbaps v. 1.1.4 (https://github.com/gtonkinhill/rhierbaps)
bacant v. 3.4.0 (https://github.com/xthua/bacant)
Beast v.2.7.6 (https://beast.community/)
TreeAnnotator v2.6.7 (https://beast.community/treeannotator)
FigTree v.1.4.3 (http://tree.bio.ed.ac.uk/software/Figtree/)
Treetime v.0.11.3 (https://github.com/neherlab/treetime)
MOB-suit v.3.1.9 (https://github.com/phac-nml/mob-suite)
Prokka v.1.14.6 (https://github.com/tseemann/prokka)
Roary v.3.13.0 (https://sanger-pathogens.github.io/Roary/)
A pipline for locating resistance genes on integrons and transposons can be found on GitHub (URL: https://github.com/tjiaa/INTS_Code).

For manuscripts utilizing custom algorithms or software that are central to the research but not yet described in published literature, software must be made available to editors and reviewers. We strongly encourage code deposition in a community repository (e.g. GitHub). See the Nature Portfolio guidelines for submitting code & software for further information.

Data

Policy information about availability of data

All manuscripts must include a data availability statement. This statement should provide the following information, where applicable:

- Accession codes, unique identifiers, or web links for publicly available datasets
- A description of any restrictions on data availability
- For clinical datasets or third party data, please ensure that the statement adheres to our policy

All complete genomic data for Salmonella is available at Bioproject number PRJNA1080262. The accession numbers of these strains are listed in Table S6.
The datasets listed below are also used in the analysis process:
pointfinder database (version 072621.2; https://github.com/guthrielab/pointfinder_db)
resfinder database (date 2023-Nov-4; https://bitbucket.org/genomicepidemiology/resfinder_db.git/src)
plasmidfinder database (date 2023-Nov-4; https://bitbucket.org/genomicepidemiology/plasmidfinder_db.git/src)
VFDB database (date 2023-Nov-4; https://www.mgc.ac.cn/VFs/main.htm)
INTEGRALL database (http://integrall.bio.ua.pt/)
THE TRANSPOSON REGISTRY (https://transposon.lstmed.ac.uk/tn-registry)

Research involving human participants, their data, or biological material

Policy information about studies with human participants or human data. See also policy information about sex, gender (identity/presentation), and sexual orientation and race, ethnicity and racism.

Reporting on sex and gender

In our study, we have carefully distinguished between the terms sex (a biological attribute) and gender (shaped by social and cultural circumstances) to avoid confusion. Our findings apply to both male and female groups, considering sex as a biological factor. The classification into male and female was based on self-reporting by participants. We have incorporated both sex and gender considerations into our study design. Participants were asked to report their sex, and methods used for this classification were based on their self-reported information. We also ensured that consent was obtained for sharing individual-level data. The source data provided in our study includes disaggregated sex and gender data wherever this information was collected. Overall numbers are provided in the Reporting Summary, ensuring transparency. If consent for sharing individual-level data was not obtained or if sex and gender data were not collected, we have explicitly stated this in the summary. We conducted sex- and gender-based analyses wherever possible. In cases where such analyses were not performed, we have provided justifications for their absence.

Reporting on race, ethnicity, or other socially relevant groupings

In our manuscript, we did not use any socially constructed or socially relevant categorization variables such as race, ethnicity, or socioeconomic status. Therefore, there were no socially constructed variables to define or categorize.

Population characteristics

In our study, we included age as a covariate-relevant population characteristic for the human research participants. Age data were collected through self-reporting by participants, with participants' ages ranging from <1 to 96 years. The patient ages are divided into 21 groups: "<1 yr", "1–4 yr", "5-9 yr", "10-14 yr", "15-19 yr", "20-24 yr", "25-29 yr", "30-34 yr", "35-39 yr", "40-44 yr", "45-49 yr", "50-54 yr", "55-59 yr", "60-64 yr", "65-69 yr", "70-74 yr", "75-79 yr", "80-84 yr", "85-89 yr", "90-94 yr", "95-99 yr", which are the same age groups adopted by the official population age statistics of the Chinese government. We did not collect or analyze other covariate-relevant characteristics such as genotypic information, past and current diagnoses, or treatment categories.

Recruitment

Our study did not involve human participants. Therefore, there were no recruitment processes or potential self-selection biases to consider. As a result, there are no biases related to participant recruitment that could impact the results of this study.

Ethics oversight | The protocols were previously reviewed and approved by the Research Ethics Committees of the First Affiliated Hospital, Zhejiang University College of Medicine (2018-752) and the Zhejiang Provincial Center for Disease Control and Prevention (2019-014).

Note that full information on the approval of the study protocol must also be provided in the manuscript.

Field-specific reporting

Please select the one below that is the best fit for your research. If you are not sure, read the appropriate sections before making your selection.

☒ Life sciences ☐ Behavioural & social sciences ☐ Ecological, evolutionary & environmental sciences

For a reference copy of the document with all sections, see nature.com/documents/nr-reporting-summary-flat.pdf

Life sciences study design

All studies must disclose on these points even when the disclosure is negative.

Sample size	We did not perform a sample-size calculation for this study. The entire sample was obtained from designated hospitals and CDC systems across 27 provincial administrative regions in China, as well as from network databases, through the Chinese Blood Bacterial Resistant Investigation Collaborative Systems. The sample size was chosen based on the availability and comprehensiveness of the data from these sources, ensuring that it is sufficient for the purposes of our analysis.
Data exclusions	In our study, data exclusions were applied to strains with sequencing quality that was too low. These exclusions were made to ensure the accuracy and reliability of our analyses. The exclusion criteria for sequencing quality were pre-established based on standard quality control measures.
Replication	All the data and software tools used in our study have been made publicly available. The main analyses were replicated more than three times independently by researchers within our laboratory to ensure the reliability and reproducibility of our findings. We confirm that using the same analysis process on the collected data yields consistent results, ensuring the reproducibility of our findings to the extent possible within the study design.
Randomization	This is not relevant to our study. We did not have control or experimental groups.
Blinding	We did not have group allocations in our study, so blinding was not necessary.

Reporting for specific materials, systems and methods

We require information from authors about some types of materials, experimental systems and methods used in many studies. Here, indicate whether each material, system or method listed is relevant to your study. If you are not sure if a list item applies to your research, read the appropriate section before selecting a response.

Materials & experimental systems

n/a	Involved in the study	
☒	☐	Antibodies
☒	☐	Eukaryotic cell lines
☒	☐	Palaeontology and archaeology
☒	☐	Animals and other organisms
☒	☐	Clinical data
☒	☐	Dual use research of concern
☒	☐	Plants

Methods

n/a	Involved in the study	
☒	☐	ChIP-seq
☒	☐	Flow cytometry
☒	☐	MRI-based neuroimaging

Plants

Seed stocks	No plants used in this study.
Novel plant genotypes	No plants used in this study.
Authentication	No plants used in this study.
